# Extramural recurrence of tracheal glomus tumour following resection by rigid bronchoscopy

**DOI:** 10.1002/rcr2.1302

**Published:** 2024-02-13

**Authors:** Makoto Takahama

**Affiliations:** ^1^ Department of General Thoracic Surgery Osaka City General Hospital Osaka Japan

**Keywords:** rigid bronchoscopy, tracheal glomus tumour, tracheoplasty

## Abstract

Glomus tumour of the trachea is very rare neoplasm that is generally benign and arises most commonly from the distal portion of the respiratory tree. This report presents the case of a 67‐year‐old man who was referred to our institute for excision of a tracheal mass that had been found incidentally, and subsequently recurred extramurally. Initial contrast‐enhanced computed tomography images of the chest revealed a nodular lesion in the trachea, 2.5 cm above the carina, that demonstrated strong enhancement similar to blood vessels. The tumour was excised by rigid bronchoscopy, but an extramural tracheal lesion was detected 18 months later. Tracheal resection and end‐to‐end anastomosis were performed, and histopathological examination confirmed the extramural lesion as recurrence of the tracheal glomus tumour. The histologic features and treatment are discussed.

## Graphical abstract

Glomus tumours of the trachea are very rare. They are generally benign and arise most commonly from the distal portion of the respiratory tree. Due to the rarity of these neoplasms, the accumulation of case details may offer some insight into how to successfully manage patients.

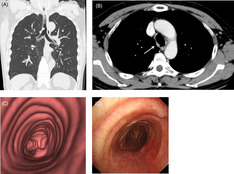

## INTRODUCTION

Glomus tumour (GT) is a rare benign tumour that originates from modified smooth muscle cells of normal glomus bodies, which form at anastomoses of arteries and veins. GT can occur anywhere in the body but most commonly affects skin. It is rarely found in the trachea, where normal glomus bodies may not be present. Due to its rarity, few data regarding tracheal GTs are available, and fewer than 80 cases have been reported.^1^ We present here a case of tracheal GT with extramural recurrence following resection by rigid bronchoscopy.

## CASE REPORT

**FIGURE 1:**
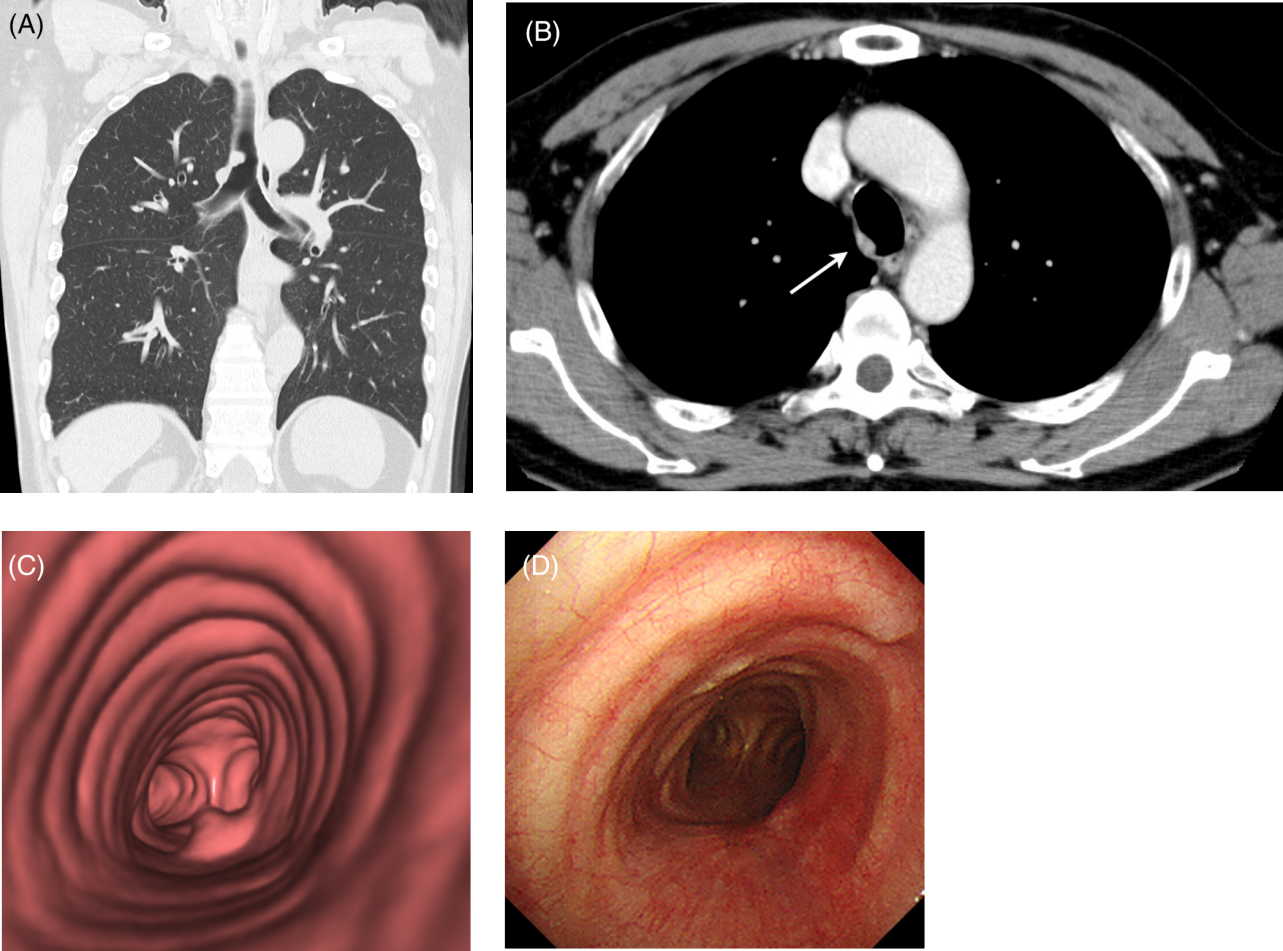
(A and B) Coronal computed tomography (CT) and virtual bronchoscope images of the chest demonstrates a 1.5 cm mass protruding into the lower trachea before endoscopic treatment. (C) The lesion shows strong contrast enhancement similar to that of blood vessels on post‐contrast axial CT before endoscopic treatment. (D) Image from bronchoscopy performed 3 months after endoscopic removal of the lesion reveals no obvious abnormalities.

**FIGURE 2:**
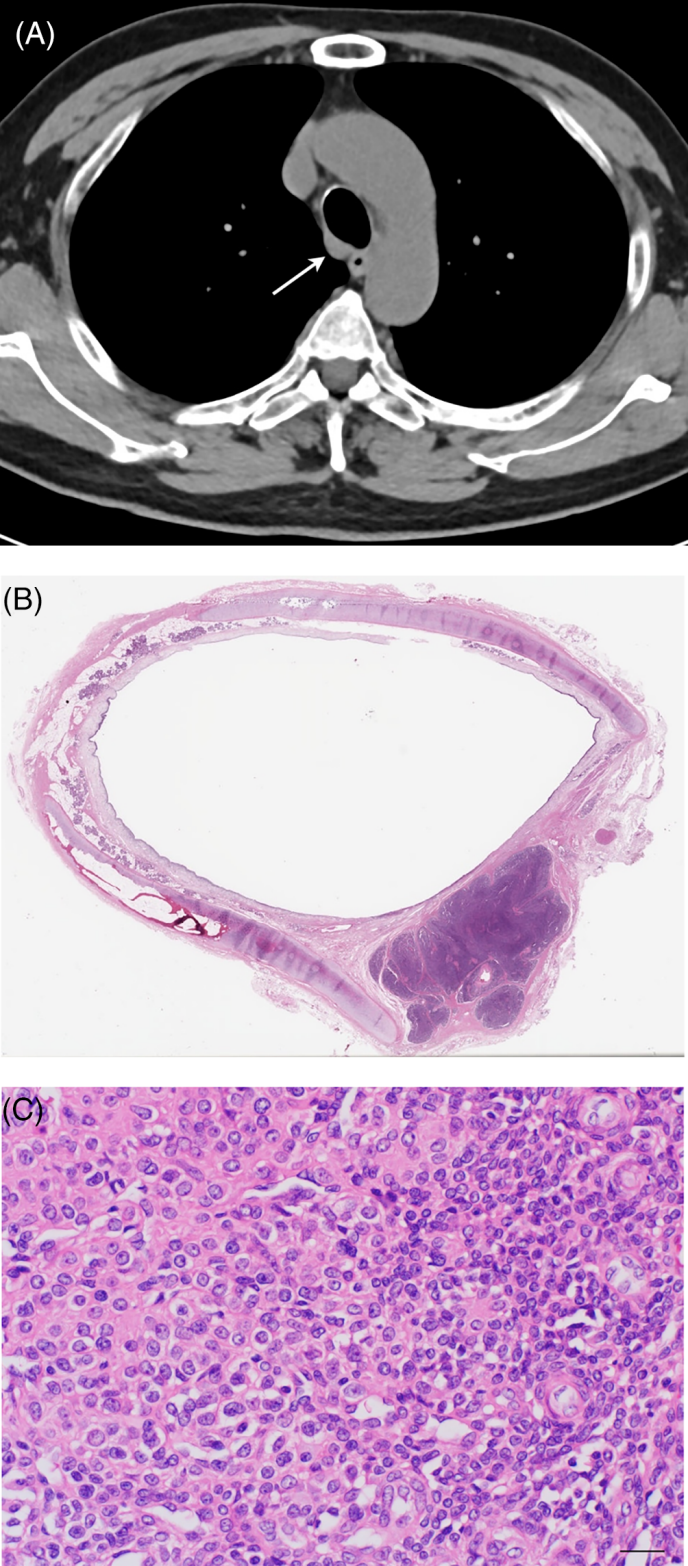
(A) Follow‐up computed tomography (CT) at 18 months after endoscopic removal of the lesion. Axial CT image of the chest shows an extramural lesion in the lower trachea. (B) Macroscopic image of the surgically resected tumour shows a 1.2 × 0.6 cm lesion with extramural protrusion. (C) Histopathological slide shows lobular arrangement of oval‐to‐spindle‐shaped cells with uniform, clear or pale eosinophilic cytoplasm and hyperchromatic nuclei. (haematoxylin and eosin, ×400). Scale bar indicates 20 μm.

A 66‐year‐old man with a 30‐year history of cigarette smoking (45 pack years) was referred to our institute following diagnosis of a tracheal mass that was found incidentally during a health‐screening check. The patient had no specific clinical symptoms and no relevant medical history. Contrast‐enhanced computed tomography (CT) and virtual bronchoscope image of the chest revealed a strongly enhancing mass lesion measuring 1.5 cm in the right posterior wall of lower trachea, 2.5 cm above the carina (Figure 1A,B). The mass showed contrast enhancement similar to that of blood vessels (Figure 1C). As the patient refused surgical resection, we performed rigid bronchoscopic resection under general anaesthesia to excise the lesion and obtain an accurate diagnosis. The patient underwent high‐frequency electrocoagulation for tumour removal. Pathologic examination of the resected tumour confirmed that the tracheal mass was consistent with GT of the trachea, which was confirmed immunohistochemically. Because the tumour was completely excised macroscopically and a postoperative bronchoscopy at 3 months demonstrated no obvious abnormalities (Figure 1D), we judged that additional treatment was unnecessary at this point. However, follow‐up CT at 18 months after the procedure identified an extramural lesion in the right posterior wall of the lower trachea (Figure 2A), which was suspected to be recurrence of the tracheal GT. As there was no definite evidence of lymphadenopathy or abnormalities in other organs, the lesion was resected surgically via right posterolateral thoracotomy. A flexible bronchoscope was used to determine the extent of resection needed. After dividing the trachea at the distal margin of the resection, we performed a wide local excision of the lesion along with four tracheal rings. Intraoperative rapid histological examination confirmed that the resection margins were free of residual tumour cells. Anastomosis was achieved by continuous suture combined with interrupted sutures using 4–0 absorbable monofilament sutures. The postoperative period was uneventful. The gross pathological findings demonstrated tumour size of 1.2 × 0.6 cm and protrusion of the tumour to an extramural site of the trachea (Figure 2B). Pathological examination found complete resection of the tumour and confirmed a diagnosis of recurrence of tracheal GT without marked nuclear atypia or any level of mitotic activity postoperatively (Figure 2C). The patient reported no symptoms and exhibited no signs of recurrence when last examined at 16 months after the surgical resection.

## DISCUSSION

GTs are composed of modified perivascular cells that resemble those of normal glomus bodies, and function to control temperature by regulating peripheral blood flow.^2^ Most GTs present as small masses in soft dermal tissues of the extremities, such as the finger subungual area, but can occur anywhere in the body. Unusual sites of origin have included the stomach, mediastinum, vagina, penis, lung and trachea.^2^ GTs of the trachea are distinctly rare. All previously reported tumours have arisen from the posterior wall of the trachea, and most were not invasive.^3^

The World Health Organization classifies these tumours into three categories: benign GTs, GTs of uncertain malignant potential, and malignant GTs.^4^ Although the majority of reported GTs were benign, a small proportion were histologically malignant.^4^ In the present case, the histological findings revealed no marked nuclear atypia or any level of mitotic activity and tumour size was less than <2.0 cm. These findings are consistent with a lack of malignant potential.^4^ However, the site of recurrence was the extramural trachea, not the tracheal location of the original portion of the tumour. This finding suggests an uncertain malignant potential due to the possibility that tumour cells could have penetrated beyond the tracheal wall.^4^

Surgery is considered the first‐choice treatment for GT. Currently available data indicate that 67.1% of reported cases were ultimately treated via surgical resection and the remainder were removed endoscopically.^2^ Endobronchial therapy can be performed in patients who are unfit for surgical resection or who refuse surgery. However, the feasibility and necessity of this method are unknown.^2^, ^3^ The possibility of tumour recurrence after endoscopic removal is higher than that after complete surgical removal, even for tumours that appear benign.^1^, ^5^ The prognosis of patients undergoing tracheal resection for GT is excellent with no reported cases of tumour recurrence.^2^ Therefore, patients with tracheal GT should be treated surgically rather than undergo endoscopic treatment.

In conclusion, tracheal GT is a rare entity that can recur after endoscopic removal, even if the tumour is benign. Therefore, surgical resection is the definitive curative treatment of choice and requires no further treatment. Endoscopic treatment may be considered for patients who refuse surgical resection.

## AUTHOR CONTRIBUTIONS

MT drafted the manuscript and approved the final version of the manuscript.

## CONFLICT OF INTEREST STATEMENT

None declared.

## ETHICS STATEMENT

The author declares that appropriate written informed consent was obtained for the publication of this manuscript and accompanying images.

## Data Availability

The data that support the findings of this study are available from the corresponding author upon reasonable request.
